# S-Doped FeOOH Layers as Efficient Hole Transport Channels for the Enhanced Photoelectrochemical Performance of Fe_2_O_3_

**DOI:** 10.3390/nano15100767

**Published:** 2025-05-20

**Authors:** Yanhong Zhou, Yiran Zhang, Boyang Jing, Xiaoyuan Liu, Debao Wang

**Affiliations:** Key Laboratory of Inorganic Synthetic and Applied Chemistry, College of Chemistry and Molecular Engineering, Qingdao University of Science and Technology, Qingdao 266042, China; wendy1061006@126.com (Y.Z.); zyr13686302960@gmail.com (Y.Z.); 13658668766@163.com (B.J.); 13908404861@163.com (X.L.)

**Keywords:** photoelectrochemicals, S:FeOOH/Fe_2_O_3_, hole transport, water oxidation, photoanodes

## Abstract

Hematite (Fe_2_O_3_) has been accepted as a promising and potential photo(electro)catalyst. However, its poor carrier separation and transfer efficiency has limited its application for photoelectrocatalytic (PEC) water oxidation. Herein, a S-doped FeOOH (S:FeOOH) layer was rationally designed and grown on Fe_2_O_3_ to construct a S:FeOOH/Fe_2_O_3_ composite photoanode. The obtained S:FeOOH/Fe_2_O_3_ photoanodes were fully characterized. The surface injection efficiency for Fe_2_O_3_ was then significantly increased with a high η_surface_ value of 92.8%, which increases to 2.98 times for Fe_2_O_3_ and 2.16 times for FeOOH/Fe_2_O_3_, respectively. With 2.43 mA cm^‒2^ at 1.23 V, the optimized S:FeOOH/Fe_2_O_3_ photoanode was entrusted with a higher photocurrent density. The onset potential for S:FeOOH/Fe_2_O_3_ cathodically shifts 70 mV over Fe_2_O_3_. The improved PEC performance suggests that the S:FeOOH layer acts as ultrafast transport channels for holes at the photoanode/electrolyte interface, suppressing surface charge recombination. A Z-scheme band alignment between Fe_2_O_3_ and S:FeOOH was deduced from the UV–Vis and UPS spectra to promote charge transfer. This method provides an alternative for the construction of photoanodes with enhanced PEC water splitting performance.

## 1. Introduction

Photoelectrochemical (PEC) water splitting using semiconductor photocatalytic electrodes has become an appealing technology for converting solar light into renewable hydrogen production to address energy-related and environmental problems [1,2]. The bottleneck of this technology is to obtain semiconductor photoelectrodes with a high photo-response and high catalytic activity to achieve efficient hydrogen production by water splitting [3,4]. To date, various semiconductor photoelectrode materials have been extensively investigated, including TiO_2_ [5,6], BiVO_4_ [7,8], α-Fe_2_O_3_ [9], WO_3_ [10], CdS [11], and so on [12]. Among numerous photocatalysts, hematite (Fe_2_O_3_) stands out as an excellent candidate for PEC water decomposition because of its moderate band-gap energy (1.9–2.2 eV), low cost, and excellent chemical stability [13,14,15,16]. However, the limited hole transmission distance (2−4 nm) and poor electron-hole carrier mobility in Fe_2_O_3_ hinder its efficient charge transfer and transport, mainly due to the significant hole recombination [17]. To address these crucial challenges, numerous studies have suggested various effective approaches, including morphology control [18,19], heterojunction construction [20,21,22,23], the incorporation of metal dopants [24,25,26,27], and cocatalyst deposition [28,29]. These approaches can effectively reduce photoanode overpotential while inducing an internal electric field, thereby improving band alignment [30,31,32,33,34].

Alternatively, FeOOH has attracted considerable attention as a promising oxygen evolution catalyst due to its environmental friendliness, non-toxicity, and abundance [35,36,37]. It has been examined as a hole transfer layer or as a passivation layer to mitigate the recombination of electron-hole pairs [38,39]. However, the low electrical conductivity of pure FeOOH remains a significant limitation that negatively affects its catalytic performance [40]. To improve efficiency, further efforts have been devoted to strategies such as metal/non-metal doping [41,42], the introduction of oxygen vacancies [43,44], and phase structure transitions [45,46]. In particular, elemental doping has been widely accepted to improve catalytic activity by modulating the electron structures of the surrounding metallic active species [47,48]. For example, Kim et al. discovered that the doping of Mn in iron oxyhydroxide (FeOOH) results in an increase in intrinsic activity by changing the electron structures of FeOOH [49]. Wang et al. stated that the surface injection efficiency of BiVO_4_ photoanodes can be significantly enhanced by growing a Ni-doped FeOOH layer on the anode [50]. Recently, He et al. reported that the doping of FeOOH with S effectively improves the charge transfer capacity of FeOOH and the surface catalytic reaction kinetics in the synthesized S-FeOOH/BiVO_4_ photoanode [51]. However, the current explanations are still unclear, and the enhancement in photocurrent signals by element doping and co-catalyst modifying requires more in-depth investigations. For example, the charge transfer mechanism between S-FeOOH and Fe_2_O_3_ remains unclear.

Herein, we report the preparation of S:FeOOH/Fe_2_O_3_ photoanodes with a sulfur-doped FeOOH layer modified on Fe_2_O_3_. The optimized S:FeOOH/Fe_2_O_3_ photoanode achieved a photocurrent density of 2.43 mA cm^−2^ at 1.23 V, showing a significant increase in surface injection efficiency to 92.8% and good stability. This performance enhancement is attributed to the influence of sulfur on the electronic structure of FeOOH, which acts as an efficient channel for hole transport at the electrode/electrolyte interface. In addition, the S:FeOOH layer reduces the onset potential by 70 mV compared to Fe_2_O_3_ when acting as oxygen evolution catalysts. Furthermore, from the results of the ultraviolet photoelectron spectra (UPS) and UV–Vis spectra, a Z-scheme band alignment between S:FeOOH and Fe_2_O_3_ has been deduced, allowing for improved charge carrier transfer.

## 2. Materials and Methods

### 2.1. Synthesis of the Photoanodes

**Scheme 1:**
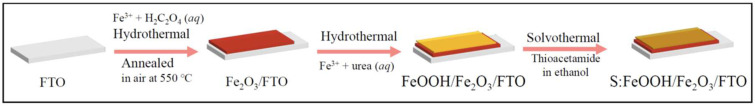
Sketch map of the synthesis procedure for the photoanodes.

The synthesis procedure for the S:FeOOH/Fe_2_O_3_ photoanodes is illustrated in Scheme 1. Firstly, a piece of fluorine-doped tin oxide (FTO) glass slide substrate (2.5 cm × 1 cm) was pre-treated by washing with acetone, ethanol, and deionized water. For the synthesis of the Fe_2_O_3_ photoanode, 0.15 mmol Fe(NO_3_)_3_·9H_2_O and 1.0 mmol oxalic acid precursor were added to 60 mL of deionized water and dissolved to form a transparent light yellow solution. It was then transferred to an 80 mL Teflon-lined autoclave and heated at 180 °C for 8 h in an oven. After natural cooling to ambient temperature, the resulting photoanode was taken out, rinsed repeatedly with deionized water and absolute ethanol, dried naturally, and finally annealed in a muffle furnace at 550 °C in air for 2 h.

For the synthesis of the FeOOH/Fe_2_O_3_ photoanode, 0.02 g Fe(NO_3_)_3_·9H_2_O and 0.01 g urea were first dissolved in 30 mL of deionized water in a 50 mL Teflon-lined autoclave. After immersing the pre-prepared Fe_2_O_3_ photoanode in the solution, the autoclave was sealed and heated at 90 °C for 90 min. After the reaction, the photoanode was rinsed three times with deionized water and absolute ethanol and then dried at 60 °C.

For the synthesis of the S:FeOOH/Fe_2_O_3_ photoanode, the pre-prepared FeOOH/Fe_2_O_3_ photoanode was immersed in 15 mL ethanol solution containing 0.3 g thioacetamide and kept at 95 °C for 3 h. After the reaction, the resulting electrode was processed in the same way as the FeOOH/Fe_2_O_3_ photoanode.

### 2.2. PEC Measurements

A CHI760E electrochemical workstation was used for all PEC measurements in a three-electrode system. The as-prepared photoanode (1 × 1 cm^2^ in size) was used as the working electrode. The reference electrode was a saturated Ag/AgCl electrode (saturated with KCl). The counter electrode was a Pt wire. Photocurrent density–potential (J-V), photocurrent density–time (J-t), and linear sweep voltammetry (LSV) curves were recorded in 1 M KOH at 1.23 V under a 300 W Xe-lamp (100 mW·cm^−2^). The cyclic voltammograms (CV) were recorded in a non-Faradaic region (0.90–1.00 V). In this research, the measured potentials were converted to RHE: V (vs. RHE) = V (vs. Ag/AgCl) + 0.197 + 0.059 pH. The EIS (electrochemical impedance spectroscopy) experiments were performed at 0.2 V vs. RHE in a frequency range of 0.1 Hz–100 kHz.

The incident photon-to-current conversion efficiency (IPCE) and the applied bias photon-to-current efficiency (ABPE) were conducted on a Modulab XM PhotoEchem workstation (Solartron Analytical, Farnborough, UK) in the 350–700 nm range with a 1.23 V bias, illuminated using a 300 W Xe lamp. The IPCE values were calculated using the following equation: IPCE (%) = 1240 × *J_ph_*/(*P* × λ) × 100%. In this equation, λ is the wavelength, *J_ph_*(λ) is the photocurrent density measured at a given wavelength, and *P*(λ) is the intensity of the incident light. The ABPE values were calculated using the following formula [52]:

(1) $$\mathrm{ABPE}\;(\%)=\frac{J\times (1.23-V_{RHE})}{P_{light}}\times 100\%$$

where *V*_RHE_ is the applied bias voltage, *J* denotes the photocurrent density at the applied bias voltage, and *P*_light_ represents the power intensity taking a value of 100 mW·cm^−2^.

The details of the material characterization are presented as the Supporting Information.

## 3. Results and Discussions

### 3.1. Material Characterization

**Figure 1:**
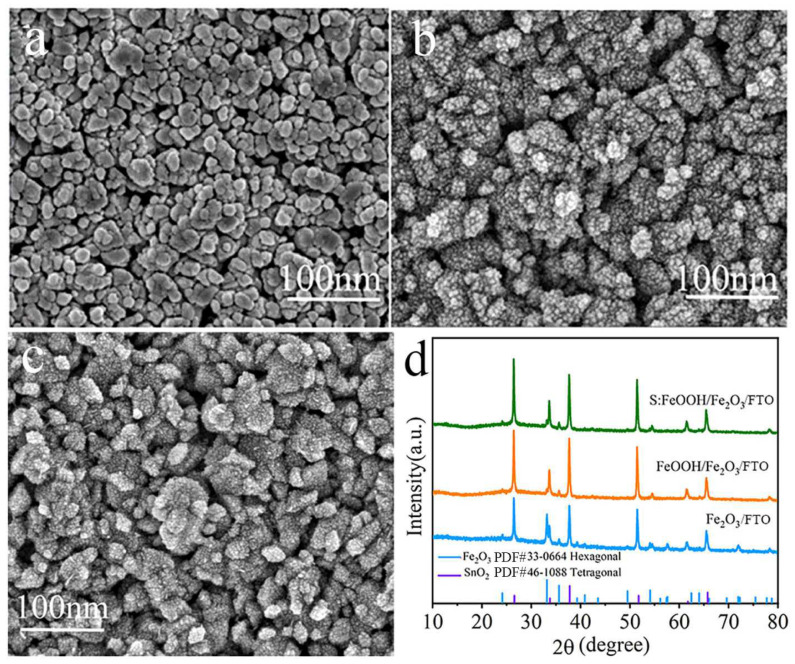
SEM images of the (**a**) Fe_2_O_3_/FTO, (**b**) FeOOH/Fe_2_O_3_/FTO, and (**c**) S:FeOOH/Fe_2_O_3_/FTO photoanodes. (**d**) XRD patterns of the prepared photoanodes.

Following the synthesis process illustrated in Scheme 1, S:FeOOH/Fe_2_O_3_ photoanodes were obtained. Figure 1a shows the SEM image of the Fe_2_O_3_ photoanode. It is obvious that a rough and porous surface rather than a dense and smooth surface was formed with irregular Fe_2_O_3_ nanoparticles. The XRD pattern of the Fe_2_O_3_ photoanode in Figure 1d indicates the presence of the hexagonal-phase hematite α-Fe_2_O_3_ according to the standard diffraction pattern in PDF#33-0664 together with the tetragonal phase SnO_2_ of the FTO (PDF#46-1088), confirming the formation of the Fe_2_O_3_/FTO composite. Subsequently, an FeOOH layer was deposited on the Fe_2_O_3_ photoanode using the hydrothermal deposition method. The SEM image presented in Figure 1b reveals the surface morphology of the FeOOH/Fe_2_O_3_ anode. After the FeOOH layer is loaded, the surfaces of the Fe_2_O_3_ nanoparticles becomes much rougher in comparison with that of the Fe_2_O_3_ photoanode. This indicates that the Fe_2_O_3_ photoanode is completely covered with the FeOOH layer through the homogenous nucleation and growth of FeOOH nanoparticles during the hydrothermal process [40]. Subsequently, S-loading was carried out via a solvothermal approach. The SEM image of the S:FeOOH/Fe_2_O_3_ photoanode is shown in Figure 1c, which indicates no obvious changes in the surface morphology in comparison to that of FeOOH/Fe_2_O_3_. However, the S-doping results in a much rougher surface for the S:FeOOH/Fe_2_O_3_ photoanode. The XRD patterns of FeOOH/Fe_2_O_3_ and S:FeOOH/Fe_2_O_3_ photoanodes are shown in Figure 1d. The diffraction peaks correspond to those of the α-Fe_2_O_3_, but no obvious peaks correspond to FeOOH, indicating the very low loading amount and/or amorphous nature of S:FeOOH.

**Figure 2:**
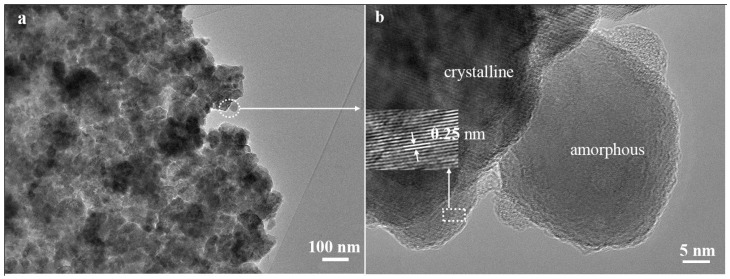
TEM (**a**) and HRTEM (**b**) images of S:FeOOH/Fe_2_O_3_.

The TEM image in Figure 2a shows a significant dark and light contrast, further indicating that the photoanode material was constructed with irregular S:FeOOH/Fe_2_O_3_ nanoparticles. The HRTEM image in Figure 2b reveals the microstructure of the S:FeOOH/Fe_2_O_3_ nanoparticles composed of crystalline Fe_2_O_3_ nanoparticles and amorphous S:FeOOH nanoparticles, indicating the formation of the FeOOH layer at the surface of the Fe_2_O_3_ anode. The lattice spacing of 0.25 nm corresponds to the (110) planes of α-Fe_2_O_3_ according to the data of PDF#33-0664, which is in agreement with the XRD results. To see the S element locations at the atomic level, the STEM HADDF image and the corresponding EDS element mappings of Fe, O, and S are presented in Figure S1. It is evident that S element homogenously distributes in the whole detection region, indicating that sulfur has been successfully doped into the S:FeOOH/Fe_2_O_3_ anode.

FT-IR and Raman spectra were recorded to further identify the composition of the electrodes. As shown in Figure S2a, the FT-IR spectra reveal the characteristic stretching vibrations of –OH groups and Fe–O bonds, as well as the Fe–OH bending vibrations. Compared to FeOOH/Fe_2_O_3_, the vibrations around 1110 cm^−1^ become significantly more serrated, corresponding to the S–O vibration [53], and the absorption bands of S:FeOOH/Fe_2_O_3_ exhibit a slight red shift. These results indicate that S^2−^ was successfully doped into FeOOH/Fe_2_O_3_ [54]. The Raman spectra of the FeOOH/Fe_2_O_3_/FTO and S:FeOOH/Fe_2_O_3_/FTO anodes are presented in Figure S2b. Both spectra show the characteristic modes of FeOOH and Fe_2_O_3_ [55]. After S doping, the Raman peaks of the S:FeOOH/Fe_2_O_3_/FTO anode become broader and slightly weaker, implying decreased particle sizes of the S-doped FeOOH/Fe_2_O_3_ nanoparticles. This further indicates that S doping altered the microstructure of the FeOOH/Fe_2_O_3_ nanoparticles [54].

**Figure 3:**
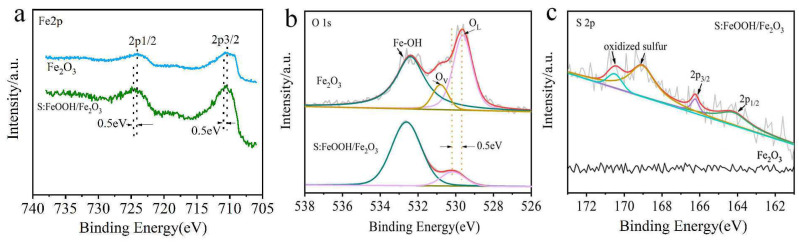
XPS spectra: (**a**) Fe 2p, (**b**) O 1s, (**c**) S 2p.

The chemical state of the relevant surface elements was characterized by means of XPS. As illustrated in Figure 3a, the high-resolution XPS spectra of Fe 2p exhibit two prominent peaks at approximately 710.8 eV and 724.4 eV, associating with Fe 2p3/2 and Fe 2p1/2, respectively. In addition, shakeup satellite peaks can be observed at around 718.2 eV and 732.9 eV. In comparison with the pristine Fe_2_O_3_, the S:FeOOH/Fe_2_O_3_ anode exhibits a positive shift of 0.5 eV in the binding energy of Fe 2p. The binding energy shift of Fe 2p after S doping indicates that sulfur should be successfully doped into FeOOH and that the electronic modulation occurs in S:FeOOH/Fe_2_O_3_ [51,56]. The O 1s spectra in Figure 3b reveals an increased peak at 532.4 eV, corresponding to surface-absorbed hydroxyl (Fe-OH) groups and a lattice oxygen peak (O_L_) around 529.8 eV. It is evident that the relative intensity of the -OH peak for S:FeOOH/Fe_2_O_3_ increases significantly. The peak area ratio of the fitted -OH peak to O_L_ peak increases from 0.69:1 for S:FeOOH/Fe_2_O_3_ to 6.1:1 for S:FeOOH/Fe_2_O_3_, thereby suggesting the successful deposition of the FeOOH layer onto the Fe_2_O_3_ film [57]. Furthermore, the O_L_ peak for the S:FeOOH/Fe_2_O_3_ sample shifts to a higher energy level, suggesting the formation of an internal electric field at its interface with Fe_2_O_3_, thereby promoting efficient charge transport across the interface of S:FeOOH/Fe_2_O_3_ [57]. Figure 3c shows the S 2p spectrum. The fitted peaks around 165 eV are assigned to S 2p_3/2_ and S 2p_1/2_ of metal sulfur bonding energy, while those around 169 eV correspond to oxidized sulfur [51]. For comparison, the S 2p spectrum for the Fe_2_O_3_ anode was also recorded and is presented in Figure 3c. It is evident that no obvious S 2p XPS signals have been recorded, which provides evidence for sulfur doping into the FeOOH layer via the solvothermal method. The presence of these S peaks provides further confirmation of the efficacy of sulfur doping into the FeOOH layer via the solvothermal method.

### 3.2. PEC Results

**Figure 4:**
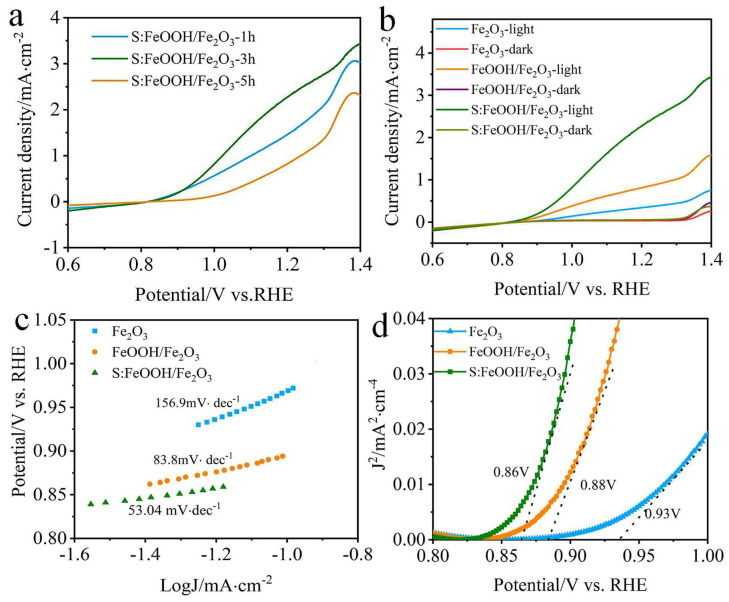
(**a**) LSV curves of S:FeOOH/Fe_2_O_3_ photoanodes with different reaction times for S doping; (**b**) LSV curves, (**c**) Tafel plots, and (**d**) Butler plots of different photoanodes.

The PEC properties of Fe_2_O_3_, FeOOH/Fe_2_O_3_, and S:FeOOH/Fe_2_O_3_ photoanodes were evaluated using 1 M KOH. The optimized reaction time for S-doping was determined to be 3 h according to the LSV curves presented in Figure 4a. The LSV curves in Figure 4b show that no obvious photocurrent was recorded at 1.23 V in the dark for any of the anodes. Under irradiation, pure Fe_2_O_3_ achieves only 0.37 mA·cm^−2^ photocurrent density at 1.23 V, which increases to 0.87 mA·cm^−2^ after the FeOOH layer is deposited on the Fe_2_O_3_ surface. The S:FeOOH/Fe_2_O_3_ anode achieves an even higher photocurrent density of 2.43 mA·cm^−2^. This represents a 5.6-fold increase over pure Fe_2_O_3_ and a 1.8-fold increase over FeOOH/Fe_2_O_3_. The Tafel plots in Figure 4c for Fe_2_O_3_, FeOOH/Fe_2_O_3_, and S:FeOOH/Fe_2_O_3_ were found to be 156.9 mV, 83.8 mV, and 53.0 mV dec^−1^, respectively. These results directly imply that the S:FeOOH layer makes a huge contribution to the water oxidation kinetics of the S:FeOOH/Fe_2_O_3_ photoelectrodes. Figure 4d displays the Butler plots (J^2^ vs. V plots) of different photoanodes. It is evident that the onset potential of Fe_2_O_3_ exhibits a cathodic shift of approximately 70 mV, decreasing from an initial value of 0.93 V to 0.86 V, indicating that the loading of the S:FeOOH layer can effectively improve the PEC activity of the Fe_2_O_3_ anode [57].

**Figure 5:**
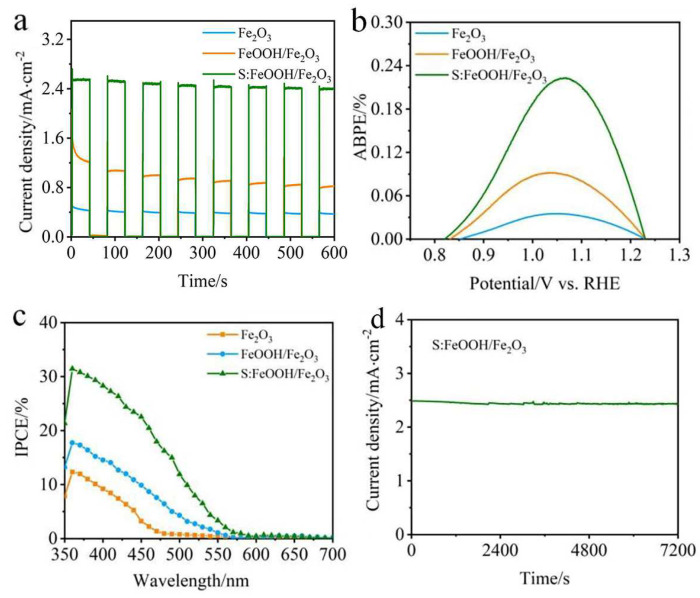
(**a**) J-t plots, (**b**) ABPEs, and (**c**) IPCEs of different photoanodes at 1.23 V vs. RHE. (**d**) Stability tests at 1.23 V under continuous illumination.

Figure 5a displays the photocurrent responses of different photoanodes. It indicates that the steady-state photocurrents during each light-on period exhibit excellent agreement with the LSV data. The ABPE plot (Figure 5b) shows that the maximum ABPE for S:FeOOH/Fe_2_O_3_ is 0.24%, which is eight times higher than the 0.03% recorded for Fe_2_O_3_. The IPCE for Fe_2_O_3_, FeOOH/Fe_2_O_3_, and S:FeOOH/Fe_2_O_3_ at 1.23 V vs. RHE is presented in Figure 5c. It shows that S:FeOOH/Fe_2_O_3_ achieves the highest IPCE value of 31.5%, exceeding the values of bare Fe_2_O_3_ (12.3%) and FeOOH/Fe_2_O_3_ (17.7%). Band gaps can be obtained from IPCE using a Tauc plot analysis of (IPCE % × hν)^1/2^ versus hν (Figure S3) in electrochemical noise mode [33]. Figure S3 shows that Fe_2_O_3_ has a band gap of 2.1 eV and the value for S:FeOOH is 1.86 eV. In addition, as shown in Figure 5d, S:FeOOH/Fe_2_O_3_ exhibits excellent stability for a period of 2 h with no decrease in photocurrent density, indicating an excellent PEC water oxidation stability.

### 3.3. Charge Transfer Kinetics

**Figure 6:**
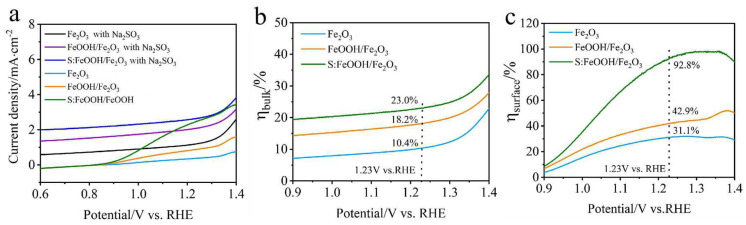
(**a**) LSV curves without and with Na_2_SO_3_. (**b**) η_bulk_ and (**c**) η_surface_ without Na_2_SO_3_ solution.

To investigate the surface and bulk charge recombination in different photoanodes, the surface charge separation efficiency (η_surface_) and the bulk charge separation efficiency (η_bulk_) were evaluated with 0.5 M Na_2_SO_3_ as a hole scavenger. Figure 6a shows the LSV curves of different photoanodes. When Na_2_SO_3_ is added to the electrolyte solution, the photogenerated holes can immediately participate in the oxidation of the water molecules arriving at the photoanode/electrolyte interface. As a result, the surface hole recombination is effectively suppressed. Due to the rapid oxidation kinetics of SO_3_^2−^ species, it can be assumed that η_surface_ is 100% for SO_3_^2−^ oxidation [34]. The η_surface_ and η_bulk_ are calculated using the equations η_surface_ = J/J_Na2SO3_ and η_bulk_ = J_Na2SO3_/J_abs_, where J_Na2SO3_ and J represent, respectively, the photocurrents with and without a Na_2_SO_3_ scavenger, respectively, and J_abs_ denotes the theoretical photocurrent density assuming that the absorbed photons completely convert into current, which is referred to as 11.4 mA cm^−2^ [58,59]. As presented in Figure 6b, the η_bulk_ values for Fe_2_O_3_, FeOOH/Fe_2_O_3_, and S:FeOOH/Fe_2_O_3_ are 10.4%, 18.2%, and 23.0%, respectively, at 1.23 V. It is evident that a significant improvement has been achieved in the bulk charge separation efficiency with the introduction of S:FeOOH. In Figure 6c, the η_surface_ values of Fe_2_O_3_, FeOOH/Fe_2_O_3_, and S:FeOOH/Fe_2_O_3_ at 1.23 V are 31.1%, 42.9%, and 92.8%, respectively. The η_surface_ value of S:FeOOH/Fe_2_O_3_ is 2.98 times and 2.66 times higher than those of the Fe_2_O_3_ and FeOOH/Fe_2_O_3_ anodes, respectively. The significant improvement can be attributed to the rapid injection of surface charges into the oxide water molecules via the S:FeOOH layer. Consequently, the S:FeOOH layer can contribute a more effective catalytic effect to the surface reactions than FeOOH alone.

**Figure 7:**
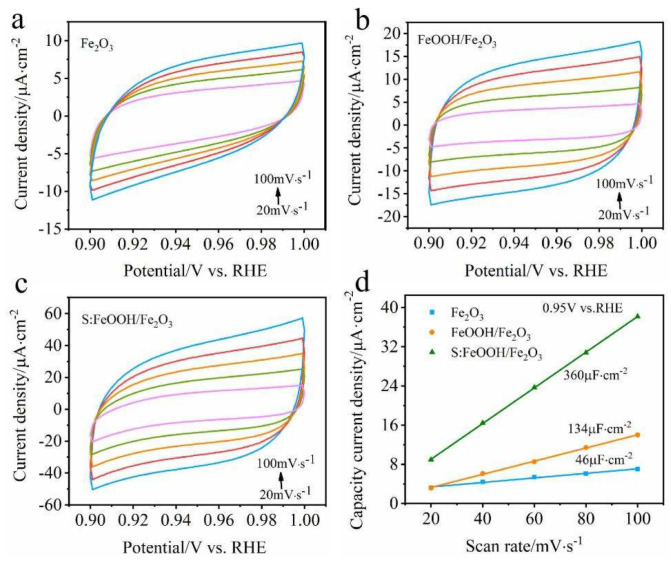
CV (**a**–**c**) and C_dl_ (**d**) plots of different photoanodes.

The cyclic voltammetry (CV) curves of different photoanodes were recorded, and the results are presented in Figure 7a–c, from which the corresponding electrochemical double-layer capacitances (C_dl_) can be deduced. As shown in Figure 7d, the C_dl_ value for S:FeOOH/Fe_2_O_3_ (360 F·cm^−2^) is significantly higher than that of FeOOH/Fe_2_O_3_ (134 F·cm^−2^) and Fe_2_O_3_ (46 F·cm^−2^). Then, the electrochemical active surface area (ECSA) of the photoanodes can be evaluated according to the proportional relationship of ECSA and C_dl_. Therefore, the S:FeOOH/Fe_2_O_3_ photoanode should have a significantly higher ECSA value, thereby increasing its intrinsic electrochemical active surface area at the catalyst/liquid interface.

**Figure 8:**
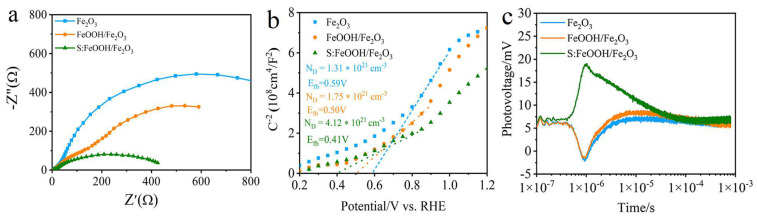
(**a**) Nyquist curves, (**b**) M-S plots, and (**c**) TPV curves for Fe_2_O_3_, FeOOH/Fe_2_O_3_ and S:FeOOH/Fe_2_O_3_.

The dynamics of the charge transfer at the electrolyte/photoelectrode interface was also examined using EIS techniques. The recorded Nyquist curves are presented in Figure 8a. It is evident that all photoanodes exhibit a characteristic arc with varying radii over the frequency range. The smaller the impedance radius, the better the conductivity and the lower the interfacial charge transfer resistance. Apparently, the S:FeOOH/Fe_2_O_3_ photoanode exhibits a minimum value of arc radii, indicating improved charge transfer performance. The observed reduction in impedance can be attributed to an increase in the internal electric field strength within Fe_2_O_3_, resulting from the introduction of the S:FeOOH layer. This enhancement promotes both electron and hole transport while simultaneously reducing surface recombination rates. Specifically, the S:FeOOH layer acts as an efficient hole transport channel, efficiently extracting photogenerated holes from Fe_2_O_3_ and accelerating the charge transfer.

Mott–Schottky (M-S) curves were carried out to further investigate the charge transfer kinetics. As illustrated in Figure 8b, the flat band potential (E_fb_) for pristine Fe_2_O_3_ is estimated to be approximately 0.59 V vs. RHE, and it exhibits a slight cathodic shift to approximately to 0.50 V and 0.41 V for FeOOH/Fe_2_O_3_ and S:FeOOH/Fe_2_O_3_, respectively. Additionally, the charge carrier density (Nd) was calculated from the M-S curve slope using the equation [60] N_d_ = (2/e_0_ɛɛ_0_)[d(1/C^2^)/dV]^−1^, where N_d_ represents the charge carrier density, e_0_ is the electron charge, ɛ is the dielectric constant of Fe_2_O_3_, ɛ_0_ is the vacuum permittivity, and V is the applied electrode potential. Therefore, the charge carrier densities were calculated to be 1.31 × 10^21^ cm^−3^, 1.75 × 10^21^ cm^−3^, and 4.12 × 10^21^ cm^−3^ for Fe_2_O_3_, FeOOH/Fe_2_O_3_, and S:FeOOH/Fe_2_O_3_, respectively. The carrier density of S:FeOOH/Fe_2_O_3_ is 2.4 times that of FeOOH/Fe_2_O_3_, proving that the introduction of the S:FeOOH layer can efficiently accelerate the formation of charge carriers, thus increasing the conductivity of the electrodes and benefiting the fast hole transport and photochemical water oxidation.

The transient photovoltage (TPV) plots for Fe_2_O_3_, FeOOH/Fe_2_O_3_, and S:FeOOH/Fe_2_O_3_ photoelectrodes are presented in Figure 8c. It is clear that both Fe_2_O_3_ and FeOOH/Fe_2_O_3_ exhibit a pronounced negative TPV signal at 1.0 × 10^−6^ s, which can be attributed to the significant charge carrier recombination [61]. In contrast, the S:FeOOH/Fe_2_O_3_ photoanode shows a significant positive TPV signal, indicating the superior separation efficiency of the photogenerated charges. This observation is consistent with the EIS results shown in Figure 8a, confirming the enhanced charge separation capabilities and reduced hole recombination of the S:FeOOH/Fe_2_O_3_ structure. Consequently, the S:FeOOH layer functions as an efficient hole transport channel between itself and the light-absorbing layer, facilitating efficient charge carrier separation.

### 3.4. Mechanism Investigation

**Figure 9:**
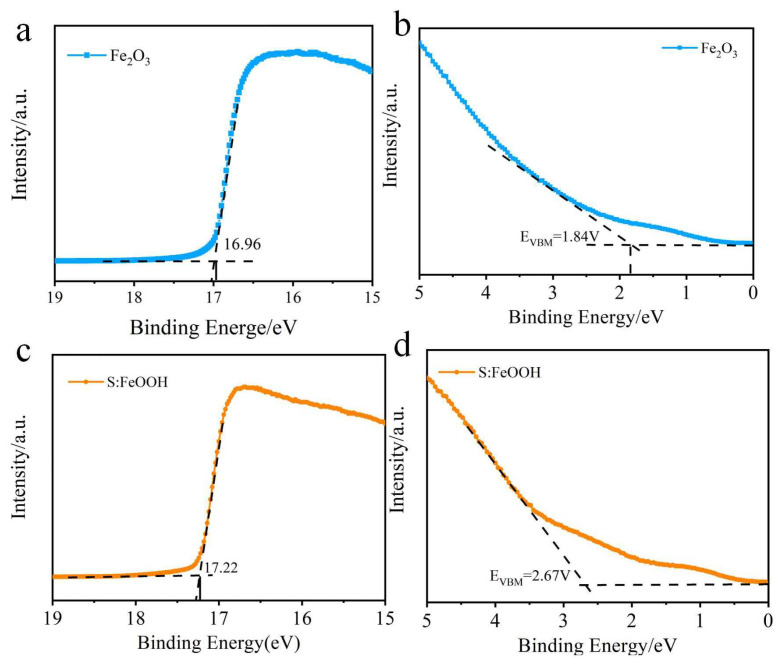
UPS for the secondary electron cut-off energy and the binding energy region of E_VBM_ of Fe_2_O_3_ (**a**,**b**) and S:FeOOH (**c**,**d**).

To investigated the PEC mechanism of S:FeOOH/Fe_2_O_3_ for enhanced PEC performance, and we analyzed the valence band structures of Fe_2_O_3_ and S:FeOOH photoanodes using UPS and UV–Vis spectra. Figures S4 and S5 present the UV–Vis absorption spectra of Fe_2_O_3_, FeOOH/Fe_2_O_3_, and S:FeOOH/Fe_2_O_3_, which show no significant changes in light absorption. This suggests that the improved photoelectrochemical performance of S:FeOOH/Fe_2_O_3_ is not due to the changes in light absorption, but rather due to other factors such as improved charge transfer and catalytic activity [51]. The energy gaps (Eg) are derived from the Tauc plots (Figure S5) from the UV–Vis absorption spectra. The results are in agreement with the Tauc plot analysis from IPCE. The secondary electron cut-off energies in Figure 9a,c are used to determine the work function, while the binding energy region shown in Figure 9b,d is used to determine the valence band maxima (VBM) for both the Fe_2_O_3_ and S:FeOOH anodes. Table S1 summarizes the values of the work functions, VBM, conduction band minima (CBM), and Eg values for Fe_2_O_3_ and S:FeOOH.

**Figure 10:**
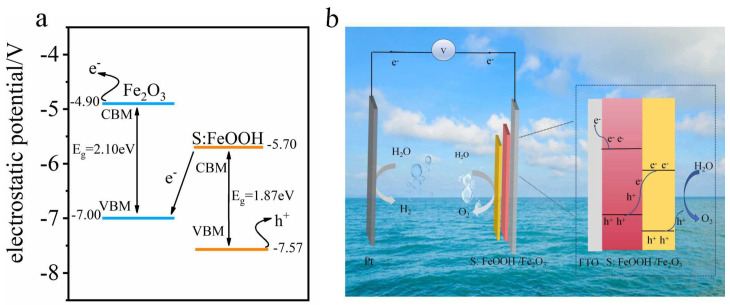
(**a**) The band structures of Fe_2_O_3_ and S:FeOOH; (**b**) a sketch map of the charge transfer mechanism for the S:FeOOH/Fe_2_O_3_ photoanode.

As shown in Table S1, the calculated band alignments for Fe_2_O_3_ and S:FeOOH exhibit a Z-scheme configuration, which is illustrated in Figure 10a, while Figure 10b schematically illustrates the charge transfer mechanism within the S:FeOOH/Fe_2_O_3_ photoanode. With illumination, electron-hole pairs are excited in both materials. Specifically, photogenerated electrons transfer from the CB of S:FeOOH to the VB of Fe_2_O_3_. Simultaneously, photogenerated holes migrate from the VBM of S:FeOOH into water to produce O_2_, and photogenerated electrons migrate from the CBM of Fe_2_O_3_ to be transported into the counter electrode to produce H_2_. This process effectively suppresses the strong surface recombination on Fe_2_O_3_ by establishing an efficient hole transport channel through the overlying S:FeOOH layer.

## 4. Conclusions

In conclusion, an S-doped FeOOH layer has been rationally designed and deposited on the surface of the Fe_2_O_3_ anodes using hydrothermal and subsequent solvothermal methods. The catalyst consists of crystalline nanoparticles and an amorphous S-doped FeOOH layer. The Z-scheme band alignment between Fe_2_O_3_ and S:FeOOH effectively increases the electron-hole pair separation efficiency. The S:FeOOH layer not only significantly increases the ECSA of the Fe_2_O_3_ anode, but also serves as an efficient hole transport channel at the electrolyte/photoanode interface, thereby enhancing the charge carrier transfer efficiency. Accordingly, the S:FeOOH/Fe_2_O_3_ photoanode achieved a significantly higher photocurrent density of 2.43 mA cm^−2^ at 1.23 V with a good stability, which is 5.6 times higher than pristine Fe_2_O_3_ and 1.8 times higher than FeOOH/Fe_2_O_3_. Its onset potential also has a 70 mV cathodic shift compared to Fe_2_O_3_. Moreover, the S:FeOOH/Fe_2_O_3_ photoanode achieves a surface efficiency (η_surface_) of 92.8%, which is 2.98 times higher than Fe_2_O_3_ and 2.66 times higher than FeOOH/Fe_2_O_3_. This simple yet effective approach offers a promising strategy for the deposition of oxygen evolution catalysts with improved performance in PEC water splitting H_2_ production.

## Data Availability

Data are contained within the article and Supplementary Materials.

## Supplementary Materials

The following supporting information can be downloaded at: https://www.mdpi.com/article/10.3390/nano15100767/s1, Material characterizations; Figure S1: HADDF image and element mappings; Figure S2: FT-IR and Raman spectra; Figure S3: Band gaps of different anodes from IPCE; Figures S4 and S5: UV-Vis absorption spectra and Tauc plots of different photoanodes; Table S1: The values of work function, E_VBM_, E_CBM_ and Eg of Fe_2_O_3_ and S:FeOOH photoanodes.
