# Supplementary material for: S-Doped FeOOH Layers as Efficient Hole Transport Channels for the Enhanced Photoelectrochemical Performance of Fe2O3
Source: Nanomaterials (Basel). 2025 May 20;15(10):767. doi: 10.3390/nano15100767 (PMC12113675; doi:10.3390/nano15100767)
Supplement: Supplementary file 1 [file nanomaterials-15-00767-s001.zip › nanomaterials-3591842-supplementary.pdf]

Electronic Supporting Information.

S-Doped FeOOH Layers as Efficient Hole Transport Channels for the Enhanced Photoelectrochemical Performance of Fe_2O_3

Yanhong Zhou, Yiran Zhang, Boyang Jing, Xiaoyuan Liu, Debao Wang*

Key Laboratory of Inorganic Synthetic and Applied Chemistry, College of Chemistry and Molecular Engi-neering, Qingdao University of Science and Technology, Qingdao 266042, China.

* Corresponding author. E-mail: dbwang@qust.edu.cn

1. Material characterizations

XRD patterns were obtained on a D/max-2500/PC X-ray diffractometer with Cu Kα radiation. SEM images were recorded using a JSM- 6700F field emission scanning electron microscope. A JEM-F210 transmission electron microscope was used to obtain TEM and HRTEM images. UV–vis diffuse reflectance spectra (DRS) were recorded using a Model C spectrophotometer fitted with an integrating sphere and BaSO4 as a reflectance standard. A Perkin Elme LS-55 spectrophotometer was used to record the PL (photoluminescence) spectra excited at a 325 nm wavelength. X-ray photoelectron spectra (XPS) and ultraviolet photoelectron spectroscopy (UPS) were recorded on a Thermo ESCALAB 50XI multi-function spectrometer with Al Kα radiation at 150 W (hν = 1486.6 eV) and He Iα photon source (hν = 21.2 eV).

FT-IR spectra were recorded on Bruker Tensor 27 IR spectrometer and the sample was prepared by the KBr pellet method. Raman analysis was performed on a LabRam HR 800 laser confocal micro-Raman spectrometer (Jobin Yvon-Horiba) with a 532 nm YAG laser as the excitation source.

2. Experimental results.

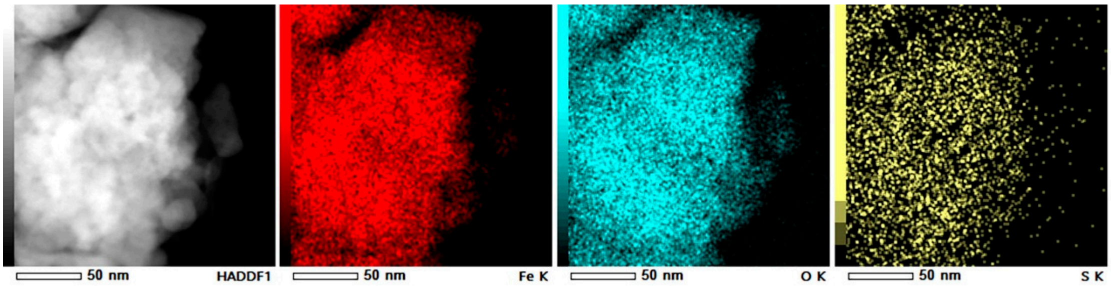

Figure S1. HADDF image and element mapping of S:$FeOOH/Fe_2O_3$.

Resolution	: 256 x 256
Instrument	: JEM-F200 (HRP)
Acc. Volt.	: 200 kV

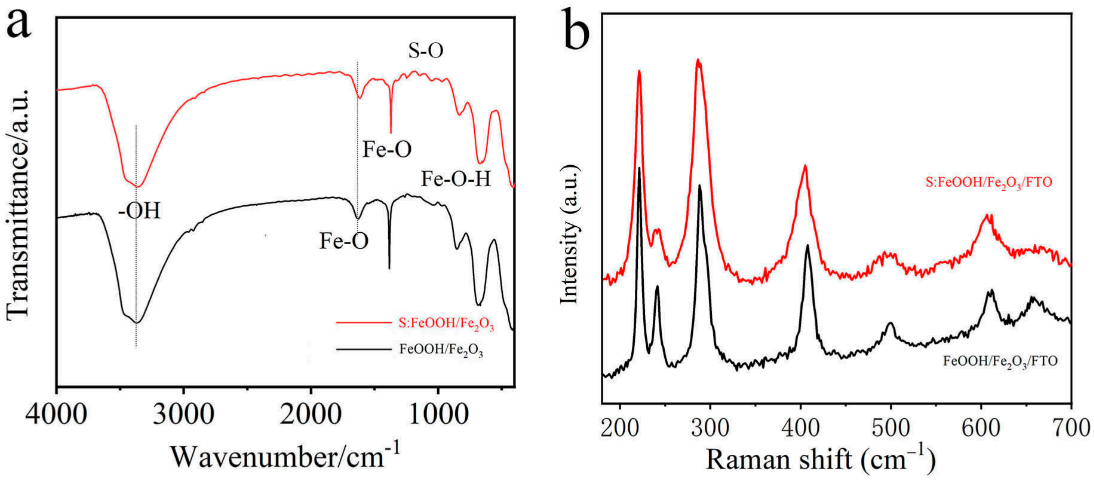

Figure S2. FT-IR spectra (a) and Raman spectra (b) of different sample.

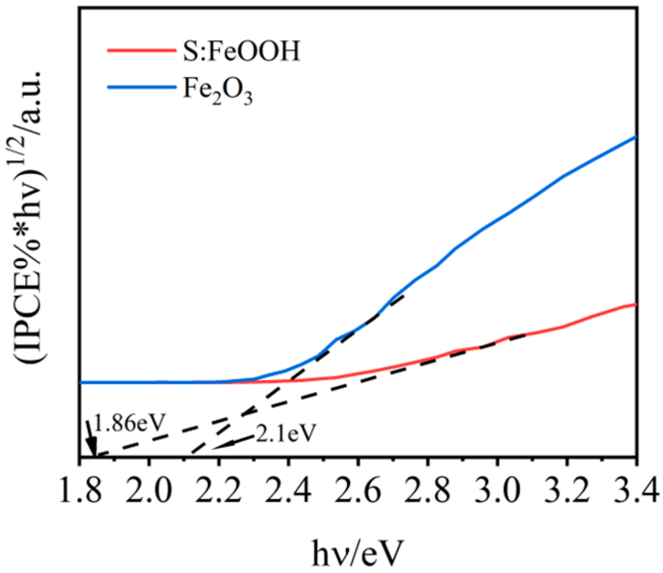

Figure S3. Band gaps of α-Fe_2O_3 photoanode and S:FeOOH photoanode based on IPCE.

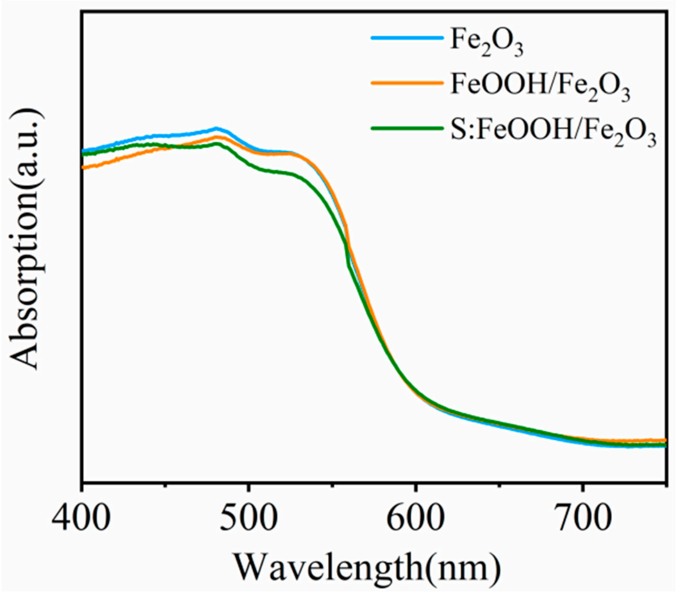

Figure S4. UV-Vis absorption spectra of different photoanodes

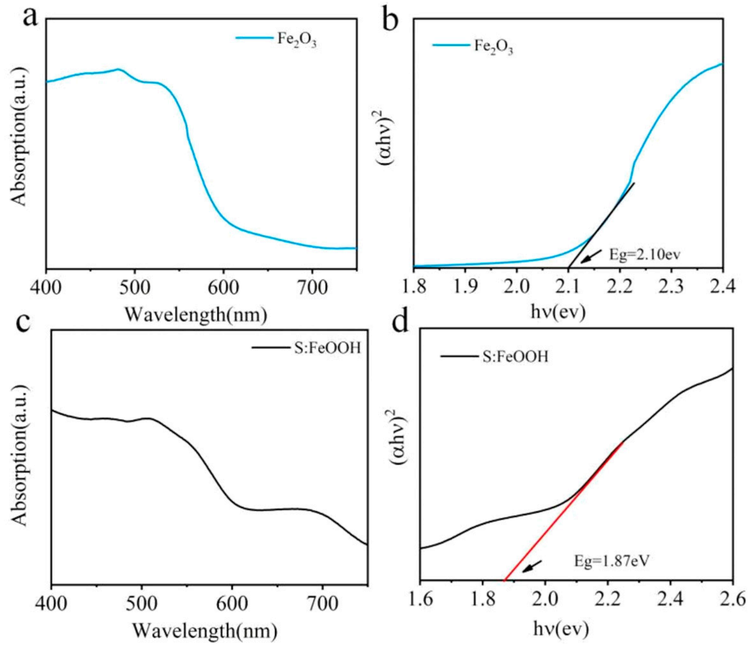

Figure S5. UV-Vis absorption spectra (a,c) and Tauc plots (b,d) of Fe_2O_3 and S:FeOOH

Table S1. The values of work function, E_{VBM}, E_{CBM} and Eg of Fe_2O_3 and S:FeOOH photoanodes.

Photoanodes	Work function (eV)	E_{VBM} (eV)	E_{CBM} (eV)	E_g (eV)
Fe_2O_3	-5.16	-7.00	-4.90	2.10
S:FeOOH	-4.90	-7.57	-5.70	1.87
